# Study protocol for improving mental health during pregnancy: a randomized controlled low-intensity m-health intervention by midwives at primary care centers

**DOI:** 10.1186/s12912-023-01440-4

**Published:** 2023-09-07

**Authors:** Marta Jimenez-Barragan, Amparo del Pino Gutierrez, Jorge Curto Garcia, Olga Monistrol-Ruano, Engracia Coll-Navarro, Oriol Porta-Roda, Gemma Falguera-Puig

**Affiliations:** 1 Fundació Assistencial Mútua Terrassa, (Terrassa), Universitat de Barcelona, Barcelona, Spain; 2 Research Group Atenció a La Salut Sexual I Reproductiva (GRASSIR), Institut Universitari d’Investigació en Atenció Primària Jordi Gol (IDIAP Jordi Gol), Barcelona, Spain; 3 Universitat de Barcelona, Barcelona, Spain; 4 Departament de Salut Pública, Salut Mental I Materno-Infantil, Facultat de Medicina I Ciències de La SalutUniversitat de Barcelona, Barcelona, Spain; 5 Ciber Fisiopatología Obesidad Y Nutrición (CIBERObn), Instituto Salud Carlos III, Madrid, Spain; 6 Patient Safety and Research Nurse, Fundació Assistencial Mútua Terrassa, Terrassa, Spain; 7 Midwifery SectionFundació Assistencial Mútua Terrassa, Terrassa, Spain; 8 Obstetrics and Gynecology Department, Hospital Universitari Mútua Terrassa, Universitat de Barcelona, Barcelona, Spain; 9 Direcció d’Atenció Primària Metropolitana Nord, Atenció a La Salut Sexual I Reproductiva Metropolitana Nord, Institut Català de La Salut, Barcelona, Spain

**Keywords:** Anxiety, Depression, Intervention, Mental health, E-health, Midwives, Mindfulness, New technologies, Pregnancy, Randomised controlled trial

## Abstract

**Background:**

Pregnancy-related anxiety and depression has received considerable attention worldwide. Mental health problems in pregnant women already since early weeks of gestation may have important consequences to the fetus. The necessity for more effective health care pathways, including some early interventions that reduce the overall burden of the childbearing situation appears a key factor for a successful birth and care of the baby. The few studies focalized in interventions, are focused on delivery and postpartum, without taking into account the whole maternity process. Current literature recommends the use of interventions based on new technologies for the treatment of mood disorders, already during the prenatal period. There have been scarce well-designed intervention studies that test technological low-intensity interventions by midwives to address pregnant women’s mental health, diminishing anxiety and depression during pregnancy.

**Methods/design:**

Adult pregnant women (weeks 12–14 of gestation) will be recruited and screened from different primary care centers in Catalonia, Spain. Women who pass the initial mental screening will be randomly allocated to the relaxation virtual reality intervention or control group. The intervention aims to improve mental state of pregnant women during pregnancy, work through breathing, mindfulness and muscle relaxation techniques. Women in the control group will receive standard care offered by the public funded maternity services in Catalonia. The primary outcome measures will include the Edinburg Postnatal Depression (EPDS), State Trait Anxiety Inventory (STAI), Symptom Checklist-90 (SCL-90), and the Cambridge Worry Scale (CWS) instruments. Secondary outcome measures will include the Temperament and Character Inventory-Revised (TCI-R) and the Whooley and Generalized Anxiety Disorder-2 (GAD-2) questions. Routinary pregnancy monitoring measures will be also evaluated.

**Discussion:**

This study aims to test the efficacy of a low-intensity, midwife-led e-health intervention based on new technologies to work on women’s anxiety and depression during pregnancy. We hypothesize that low-intensity mental health intervention during pregnancy, using an e-health (virtual reality) as a support tool, will be effective in reducing of anxiety, depressive symptoms, and improving satisfaction with pregnancy follow-up.

**Trial registration:**

Clinical Trials ID NCT05756205.

**Supplementary Information:**

The online version contains supplementary material available at 10.1186/s12912-023-01440-4.

## Background

Motherhood is a normal process of life characterized not only by physiological and biological changes in the mother's body, including physical brain changes, but also by a psychological adaptation to the new reality of pregnancy, childbirth and the future baby [1–5].

The prevalence of anxiety and depression in pregnant women has significantly increased after the spread of coronavirus-19 (COVID-19) throughout the world. Anxiety related to pregnancy is relatively common, with a current prevalence COVID-related between 26%-57% of women, appearing to be a unique syndrome reflecting fears about the health and well-being of self and one’s baby, of the hospital and of impending childbirth, and of parenting or the maternal role [6].

Depression during pregnancy shows a worldwide antenatal prevalence of 9% [7], increasing during COVID period from 20 to 31% [8]. In some populations these percentages of anxiety and depression are as high as 65% and 56%, respectively [9]. In addition, these disorders can also manifest jointly [10, 11]. Importantly, both anxiety and depression in pregnancy are associated with postnatal depression and poor maternal–infant bonding after childbirth [12, 13].

Likewise, the negative impact that anxiety and depression during pregnancy can have on both mother and child, due to the increase in the hormone cortisol, has also been demonstrated [14–17]. Some prospective studies relate maternal anxiety to adverse neurodevelopmental outcomes, including cognitive, emotional and behavioral problems [15, 18]. The literature has also associated prenatal anxiety with low gestational weight infants, premature births and arterial hypertension [18].

Pregnancy-related anxiety has also been associated with an increased number of follow-up visits [19], increased tobacco and alcohol consumption [20, 21], and increased likelihood of having a cesarean section and requesting an elective cesarean section [22–24]. Moreover, women who manifested anguish during maternity were more likely to experience a higher risk of experiencing disaffection towards their children and postpartum depression [11, 25]. These factors are especially relevant because they have a negative influence on pregnancy, childbirth and the postpartum period, and affect the way in which women face motherhood and their new role as mothers.

In a very recent study of our group assessing the prevalence of anxiety and depression and their associated risk factors throughout the pregnancy and postpartum process using a new screening for the early detection of mental health problems, we found that the most relevant factors associated with positive screening for antenatal depression or anxiety during pregnancy, that appeared from the first trimester of pregnancy, were systematically repeated throughout the overall gestation, and were maintained in the postpartum period. This results in a sustained impact of anxiety and depression as early as the first weeks of gestation. The result of early screening for anxiety and depression in pregnancy suggests the need for more effective health care pathways, including some early interventions that reduce the overall burden of the childbearing situation, both on the mother herself, and her child consequently, and on the health care system.

### Existing interventions to help women with mental health problems during pregnancy

Despite the high burden of psychiatric disorders affecting pregnancy and childbearing, there is little empirical research on the most effective interventions to reduce it [11, 14]. In this scenario, the treatment of psychiatric disorders during pregnancy becomes even more important, due to all its consequences, given the great increase that has occurred in recent years due to the COVID pandemic.

The few interventional studies are focused on delivery and postpartum, without considering the whole maternity process (pregnancy, delivery, and puerperium) [14, 15]. In this sense, the ideal intervention is in the detection, assessment, and support during the entire maternity process, starting in early pregnancy stage [26]. Likewise, the support of the midwife, the quality of the relationship established with the woman and the level of her involvement in the choices that affect her, empower the pregnant woman, and increase her satisfaction in the maternity process [27].

The National Institute of Clinical Excellence (NICE) guidelines describe two levels of intervention for women with mental health problems: high-intensity interventions to be carried out by a specialized professional, and low-intensity interventions that can be provided by a facilitator, and where self-help materials can be used [26]. One or other type of interventions will be determined according to the problem and/or the mental diagnosis. Therefore, the midwife, within the multidisciplinary team, has a fundamental role not only in the detection of women presenting a perinatal mental health problem, but also in the support and empowerment of these women through low-intensity interventions.

Different types of low-intensity interventions to reduce anxiety during pregnancy have been described. These interventions, through the relaxation of the body and the mind, such as hypnotherapy, yoga, meditation and mindfulness, are considered effective in changing the perception of anxiety during pregnancy. With them, the woman can change the perception of a stressful situation, developing better adapted coping strategies [28]. Different authors conclude that mind and body interventions could be useful for prevent and treat anxiety during pregnancy without adverse effects [28, 29]. However, the literature recommends investigating them with experimental studies, adequate sample measurements and the use of valid tools to measure their impact in the perinatal period [28].

Within this scenario, there are new technologies under development which are capable of promoting positive changes in people's health [30]. *E-health* is a tool that puts technology, informatics and the Internet at the service of health; and *m-health* is defined as the use of mobile, phones and other wireless technology in medical care in a direct, low-cost and engaging manner [31–34]. Their use is growing exponentially all over the world. Current literature recommends the use of *m-health* interventions for the treatment of mood disorders, and emphasizes the need to conduct new research on the results of these interventions in the prenatal period [35].

### Aims

To test the efficacy of a low-intensity intervention led by midwives to improve mental well-being during pregnancy, using an *e-health* application as a support, in primary care centers.

## Design and methods

This will be a two-arm prospective, randomized, parallel-controlled clinical trial. 70 women will be recruited and screened during their pregnancy. Women showing positive scores in an early mental health screening at weeks 12–14 of pregnancy (screening tests: two Whooley questions, two Generalized Anxiety Disorder-2 [GAD-2] questions, self-administered Edinburg Postnatal Depression [EPDS]) will be randomly allocated to an intervention or control group.

If the answer to all four initial questions is negative, we will consider the screening negative. If the answer is positive to any of the four questions, the screening is positive, and it is recommended to administer the EDPS. EDPS is negative if score < 13 or item 10 is negative. EPDS is positive if score < 13 and item 10 positive or ≥ 13. If the answer is positive to any of the four questions, but the EDPS is between 9–13, they are candidates to enter the study.

The usual monitoring of pregnancy control will be carried out at the center (including anamnesis, blood tests, serology and urine tests, among other assessments), regardless of the group. In a first phase (weeks 12–14 of gestation), the following tests will be performed in both groups: EPDS, State Trait Anxiety Inventory (STAI), Symptom Checklist-90-Revised (SCL-90-R), and Temperament and Character Inventory-Revised (TCI-R). The intervention group will receive the usual mentioned follow-up pregnancy monitoring plus an *e*-*health* (virtual reality) intervention. The control group will receive the usual mentioned follow-up pregnancy monitoring, without the *e*-*health* intervention. In a second phase (6 weeks after), the following assessments will be repeated in both groups: EPDS, STAI, and SCL-90. In the case of the intervention group, a questionnaire on the usability of the technology will also be passed. At the end of the pregnancy all women will receive a questionnaire on their satisfaction with the follow-up of their pregnancy, including questions on equipment, accessibility, organization of the consultation and care and competence of the staff (28 questions in total). Providing a control group that receives the same monitoring control establishes whether the technological low-intensity intervention accounts for the results. The involvement of midwives in collaboration with mental health nurses will maximize the translation of research findings into practice.

### Hypothesis

Low-intensity mental health intervention during pregnancy, using *e-health* as a support tool, is effective in reducing of anxiety, depressive symptoms, and improving satisfaction with pregnancy follow-up.

### Outcome measures

The primary outcome measure is possible mental health improvement in pregnant women according to the EPDS, STAI, and SCL-90. Secondary outcome measures will include the TCI-R and the Whooley and GAD-2 questions. Routinary pregnancy monitoring measures will be also evaluated (i.e., anamnesis, blood tests, serology, and urine tests).

### Setting

Primary care centers of the Sexual and Reproductive Health Care (ASSIR; *Atenció a la Salut Sexual i Reproductiva*) of Mutua Terrassa, Barcelona (Spain), with approximately 1,700 pregnancies per year.

### Sample and sample size

Pregnant women ≥18 years followed in the primary care centers of the Sexual and Reproductive Health Care of Mútua Terrassa, Barcelona (Spain) will be invited to participate. 

There are very few scientific publications on which to base our assumptions about the differences that the intervention will produce in the results of the scales that measure anxiety in pregnant women. In a previous interventional work published using a mindfulness program to evaluate changes in the EDPS scale in pregnant women, the authors report after the program a mean difference between the two groups of study (intervention and control) of -2.56, with a standard error (SE) of 0.72 [36] From that, the standard deviation (SD) has been calculated as: SE=SD/Root(n), so SD=SE*Root(n). In this case, as the n was different in both groups (51 and 45), we use the minimum of both (45), obtaining a joint SD of 4.83. 

Assuming a power of 80% to detect differences in the contrast of the null hypothesis H_0_: μ_1_=μ_2_ by means of a bilateral T-Student test for two independent samples, a significance level of 5%, and assuming a mean difference between both groups of -3.5 and a SD of both groups of 4.83 units, it will be necessary to include 31 women in the non-intervention group and 31 women in the intervention group, totaling 66 patients in the study. Considering that the expected drop-out rate is 5%, it would be necessary to recruit 70 women (35 in each group). 

### Inclusion criteria

All women who control their pregnancy in the primary care centers of the Sexual and Reproductive Health Care (ASSIR; *Atenció a la Salut Sexual i Reproductiva*) of Mutua Terrassa, Barcelona (Spain) will be invited to participate. The ASSIR is a support structure for primary care and is located between primary and specialized care, with the aim of promoting, encouraging and coordinating activities for comprehensive sexual and reproductive health care for women. The women must present a positive value in the mental health screening performed at the beginning of the pregnancy (weeks 12–14 of gestation). Women scoring positive (EPS 9–12) will be randomized to either the control or intervention group. The women must be able to have a good verbal and written understanding of Spanish and be ≥ 18 years old.

### Exclusion criteria

Women with diagnosed psychiatric pathology who are already being followed by the mental health team will be excluded from the study. Women victims of gender-based violence who tested positive in the partner violence screen (PVS) were also excluded [37].

### Recruitment protocol and randomization procedure

The research will follow a pre-established recruitment protocol following ethical guidelines for human research. The selection of the women will be done consecutively, during the visit 12–14 months of gestation. The staff will be explained on the project an invite eligible pregnant women to contact to the research midwife. After obtaining informed consent, women will be invited to provide contact details and complete an initial screening plus a full questionary about different sociodemographic and clinical aspects. Women who will answer "yes" to any of the four questions of the screening test, and who in the EDPS will score between 9 and 12, will be candidates for the study. The questionnaire responses will then be evaluated and the women will be contacted and, with their permission, randomly assigned to one of the two treatment groups. The principal investigator will create a randomisation list with the computer epidemiological program EPIDAT (Epidat 4.2) after participant’s written consent and not blinded to the woman’s allocation.

Randomisation of the study subjects will be masked. Women who enter the study will be scheduled for an extra visit with the midwife between weeks 15 and 17 of gestation. The woman who will be assigned to the control group will be informed that the follow-up will be as usual. The women in the interventional group will receive information about the intervention using *e-health* support (immersive virtual reality).

Participants will also be provided with a contact email address if they require additional support.

### Measures

*Edinburg Postnatal Depression Scale (EPDS)* [38]*.* This self-reported scale consists of 10 items. The participant chooses which of the four possible answers most closely resembles how she felt the previous week. The validity of the scale into Spanish is satisfactory, with a sensitivity of 79% and a specificity of 95.5%. It showed also good reliability with a Cronbach's alpha of 0.87. The scores range between 0 and 30, and results > 13 or < 13 but item 10 positive during pregnancy are considered positive.

*Generalized Anxiety Disorder-2 (GAD-2)*. This is a very quick screening, comprised by two questions strongly related to anxiety disorder symptoms: “Have you felt nervous, anxious or overwhelmed?” and “Have you felt unable to control or stop your worries?”. For this study, answers to each item will be yes/no. This screening tool has shown a good level of sensitivity and specificity in different studies of validation. If the answer is positive to any of the questions (including the Whooley questions, see below), the screening will be positive, and it will be recommended to administer the EDPS.

*State Trait Anxiety Inventory (STAI)* [39]*.* The aim of the STAI questionnaire is to evaluate two independent concepts of anxiety, each with 20 questions. The state dimension evaluates a transitory emotional state, characterised by subjective, consciously perceived feelings of attention and apprehension and hyperactivity of the autonomic nervous system. The trait dimension evaluates an anxious, relatively stable propensity that characterises individuals with a tendency to perceive situations as threatening. The clinical validity of the Spanish scale showed a sensitivity of 0.94 and a specificity of 1. The internal consistency showed a Cronbach's alpha index between 0.83 and 0.90.

*Symptom Checklist-90-R (SCL-90-R)* [40]*.* Questionnaire made up of 90 items that describe a specific psychopathological or psychosomatic disorder. It is divided into somatization, obsession-compulsion, interpersonal sensitivity, depression, anxiety, hostility, phobic anxiety, paranoid ideation, psychoticism and an additional scale. The intensity of each symptom has to be graduated by the subject from 0 (absence) to 4 (maximum annoyance). The evaluation of the Spanish version showed an internal consistency with Cronbach's alpha index of 0.81–0.90.

*Temperament and Character Inventory-Revised (TCI-R)* [41]. This inventory measures 7 dimensions of personality, composed of 4 of temperament (avoidance of harm, novelty seeking, reward dependence and persistence) and 3 of character (self-direction, cooperation and transcendence). It consists of 240 items measured on a 5-point Likert scale. The Spanish version has shown satisfactory psychometric properties, with a Cronbach's alpha ranging from 0.77 to 0.84.

*Usability Evaluation APP-M-health* [42, 43]*.* An abbreviated, translated and modified version, derived from the *'Questionnaire d'expérience utilisateur*' of the Tcha-Tokey et al. (2018) questionnaire has been used. This adapted version can be found in Additional file 1.

*Whooley questions*. This screening consists of two dichotomous questions, as in the case of GAD-2 screening: “Have you ever felt low spirits, depressed or hopeless?” and “Have you often felt that you have lost interest or pleasure in things?” (Answer: yes/no). As mentioned above, if the answer is positive to any of the questions (including the GAD-2 questions), the screening will be positive and it will be recommended to administer the EDPS.

*Pregnancy satisfaction questionnaire* [44]. It is a validated self-completed scale with 28 items that assesses aspects related to four dimensions: equipment, accessibility, organization of the consultation and care and competence of the staff. Each question is measured on a 5-point Likert scale. The questions are written as form that some of them express positive aspects and others negative. Overall satisfaction is defined as the sum of the scores awarded to each of the judgments, divided by 28. A woman is considered satisfied with the follow-up of her pregnancy if she obtains an overall satisfaction > 3, and clearly satisfied if the score is > 4. Cronbach's alpha was 0.92, ranging from 0.71 to 0.93.

### Control group

Pregnant women in the control group will be cited for an extra visit, where they will be given the following scales: STAI, SCL-90 and TCI-R. These women will undergo the usual monitoring of pregnancy control at the center by the midwife, according to the Protocol for Monitoring Pregnancy of the Generalitat de Catalunya [45]. The women will be called to back after 6 weeks to go through the same scales again (except the TCI-R), and the pregnancy satisfaction questionnaire.

### Interventional group: *m-Health*

**Fig. 1:**
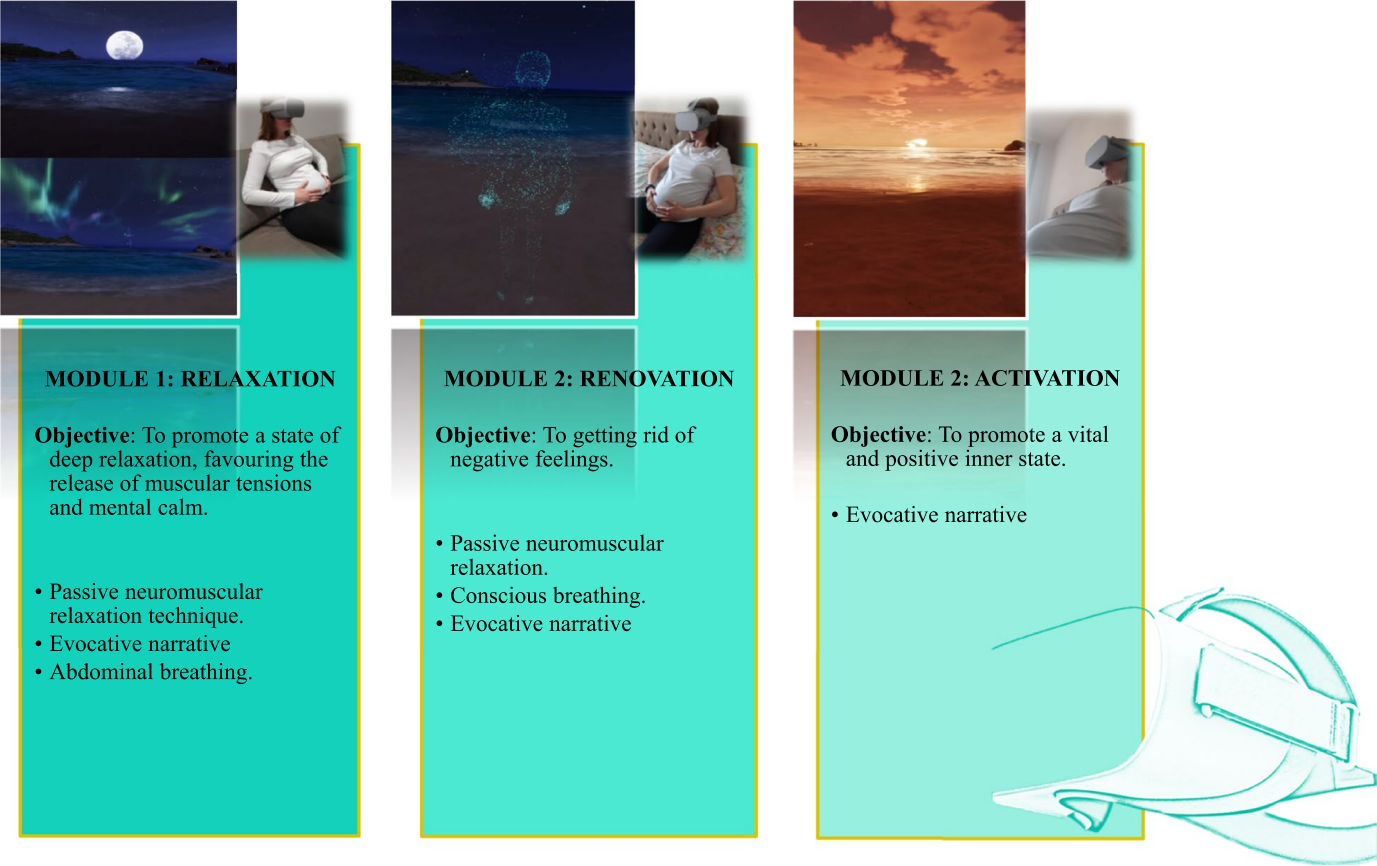
Modules forming the *m-health* virtual reality intervention

An *e-health* intervention that will not require advanced qualifications by the nurses involved in the study has been developed. The intervention will consist of an Immersive Virtual Reality (IVR) application with virtual reality goggles Oculus GO, to reduce anxiety during pregnancy. This application lasts 14 min by the use of mindfulness techniques based on breathing, mindfulness and passive muscle relaxation. It consists of three modules that can be chosen completely or separately (Fig. 1).

The intervention will occur off site using the wireless technology. The aspects that conform this intervention will be: information about the most common perinatal mental health problems, exercises based on attention to breathing (mindfulness—relaxation), 6 weeks of duration, for 14 min a day, knowledge from involved personnel of the number of times the woman has connected and the time she has done the exercise, satisfaction questions to pregnant women after pregnancy, alerts to notify the principal investigator if there is a problem with *e-health*, and, finally, an email address to contact the principal investigator. This intervention has been previously evaluated by a committee of eight experts. These experts gave their recommendations to improve the virtual application. Once the changes were applied, a pilot test was conducted with five women who reported the level of ease of viewing, using and manipulating the application.

Specifically, the virtual application will consist of an adaptation to pregnant women of an application for the general public. The main character that appears in the application is a female avatar, who talks about questions aimed at the target group of pregnant women.

### Consent

Eligible women will be given written information outlining the study purpose, invited to ask questions, and be able discuss their participation with family or care providers.

An informed consent will be obtained from all the participants that agree to participate. Participants will be advised they can withdraw from the study at any time without effect to their care. Women will also be provided with contact details for the research team.

### Data collection and management

**Fig. 2:**
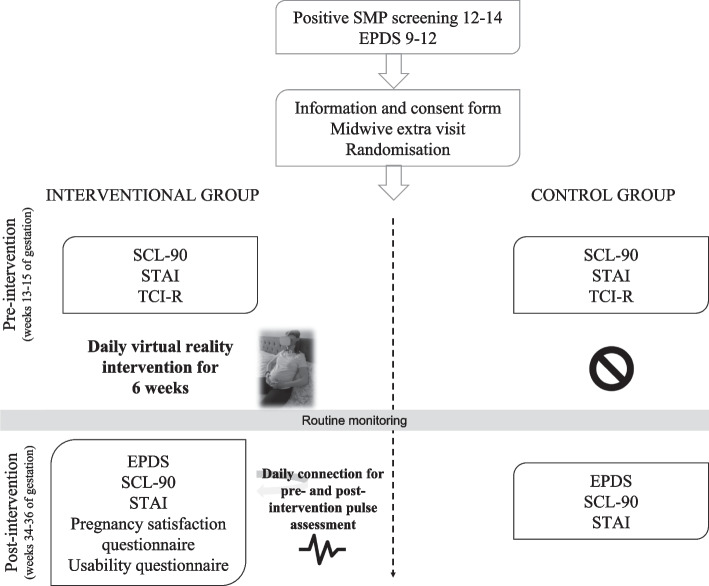
Study design: assessments in the control and intervention groups. Abbreviations: EPDS, Edinburg Postnatal Depression; SCL-90, Symptom Checklist-90; STAI, State Trait Anxiety Inventory; TCI-R, Temperament and Character Inventory-Revised

Data collection is shown in Fig. 2. Data will be collected at 3 time points; Recruitment 12–14 weeks of pregnancy, pre-intervention and post-intervention. Data will be stored in line with the Mútua of Terrassa ethical procedures considering data storage and security procedures. Participants’ questionnaires will be coded with a numerical identifier and kept separately from any identifiable personal information such as names or addresses.

## Data analysis

### Quantitative data analysis

Data analysis will be primarily descriptive. Participant flow through the study will be presented following Consolidated Standards of Reporting Trials (CONSORT) guidelines [46]. Descriptive data will be presented by means and SDs; medians and ranges; or percentages with 95% confidence intervals (CIs), as appropriate depending on the data being described. The following will be calculated: (1) percentage of participants meeting eligibility criteria, (2) percentage of individuals consenting to the study, (3) percentage entering the randomisation phase, (4) the number of sessions completed by those in the treatment arm, (5) percentage completing the outcome measures at post-treatment follow-up, (6) between group pre-post effect sizes and CIs on the mental health tests. T-tests, Mann Whitney U tests, and or Wilconxon tests will be used, as appropriated. Quantitative analyses will be performed with the program Statistical Package for the Social Sciences (SPSS) v23.0 (IBM company, Chicago, Illinois, USA). A p < 0.05 will be considered statistically significant.

### Ethical approval

The research protocol has been reviewed and received approval from the Drug Research Ethics Committee (CEIM; Comité de Ética de la Investigación con medicamentos, CEIM) from Mútua Terrassa (02/2018). The study will be conducted in accordance with the principles of the Declaration of Helsinki (World Medical Association Declaration of Helsinki, 2001) and its later amendments (revised in 2013), and according to the Regulation UE2016/679 of the European Parliament and of the Council of 26 of April de 2016 on the protection of individuals regarding the processing of personal data and the free movement of data.

## Discussion

This proposal addresses a pioneering low-intensity intervention through IVR focused on pregnant women with anxiety and depression problems to improve their mental quality, from the initial stage of pregnancy. This proposal will be carried out in different primary care centers in Catalonia, Spain. The protocol tests the effectiveness of an evidence-based intervention (*e*-*health*), in this case led by midwives and specifically designed in this study for pregnant women, to improve maternal and perinatal mental health problems during pregnancy. The key outcome is to optimise the mental health of pregnant women, promoting normal childbirth and indirectly reducing the burden on the health system.

The proposed protocol evaluates an innovative program that integrates evidence and is feasible in clinical practice. First, it uses a proven intervention in general samples that combines evidence-based strategies with comprehensive knowledge of mood disorders to provide mental health support [32, 35]. Second, it offers home-based intervention, thereby improving accessibility, timeliness, and flexibility for pregnant women. Third, the intervention does not focus on psychopathology and mental problems per se; but strengthens and supports women’s peace of mind, through mindfulness techniques based on breathing, mindfulness and passive muscle relaxation. And fourth, but not least, this intervention is led by midwives, which makes it interesting, as it reduces the health pressure on other professionals to deal the mental problems of pregnant women in the early stages of pregnancy, improving their overall well-being, and requiring less medical attention and referrals to specialists for these issues. The results of this study will contribute to reducing anxiety and depression in pregnant women, in a very simple way, by promoting effective emotional care in the prenatal period by midwives.

What this study can bring to the treatment of anxiety and depression in pregnant women using this virtual reality technology will help to improve the overall therapeutic approach to pregnant women, so that this low-intensity intervention can be incorporated systematically into primary care consultations. Addressing mental health in pregnant women is a fundamental approach to preserve a peaceful pregnancy, free of stress and mood disorders that can affect the fetus and its birth, thus reducing postpartum problems for both the child and the mother and complications for normal infant care.

All this leads us to speak of an increase in the quality of life (QoL) of pregnant women, and of their postpartum period, providing strategies that can help them not only during pregnancy but also afterwards. All this will always translate into an improvement in the upbringing of their children.

The study is expected to take three years. The first two months will be required to hire and train staff. Recruitment will then commence and continue for 24 months. In year 3 we will complete data analysis, reporting and prepare publications and refine resources developed in the project.

In summary, we consider that our project provides very innovative elements that can improve QoL of pregnant women, providing mental resources for their future as mothers. The use of new technologies (IVR) also makes this initiative an attractive project and easy to implement in the future, through the platform used or others. The involvement of midwives in collaboration with mental health nurses will maximize the translation of research findings into practice.

### Supplementary Information

**Additional file 1.** Immersive Virtual Reality (IVR) to reduce anxiety during pregnancy.

## Data Availability

Not applicable.

## Abbreviations

ASSIR: *Atenció a la Salud Sexual y Reproductiva* (Sexual and Reproductive Health Care)
COVID-19: Coronavirus-19
EPDS: Edinburg Postnatal Depression
GAD-2: Generalized Anxiety Disorder-2
IVR: Immersive Virtual Reality
QoL: Quality of Life
STAI: State Trait Anxiety Inventory
SCL-90: Symptom Checklist-90
SD: Standard Deviation
SE: Standard Error
TCI-R: Temperament and Character Inventory-Revised
