# Supplementary material for: Study protocol for improving mental health during pregnancy: a randomized controlled low-intensity m-health intervention by midwives at primary care centers
Source: BMC Nurs. 2023 Sep 7;22:309. doi: 10.1186/s12912-023-01440-4 (PMC10483870; doi:10.1186/s12912-023-01440-4)
Supplement: Supplementary file 1 — Additional file 1. Immersive Virtual Reality (IVR) to reduce anxiety during pregnancy. [file 12912_2023_1440_MOESM1_ESM.docx]

Additional file 1

Immersive Virtual Reality (IVR) to reduce anxiety during pregnancy

*User experience questionnaire*

| 1 – muy de acuerdo  5 – nada de acuerdo | **Valoración (1-5)** | | | | |
| --- | --- | --- | --- | --- | --- |
|  | **1** | **2** | **3** | **4** | **5** |
|  | **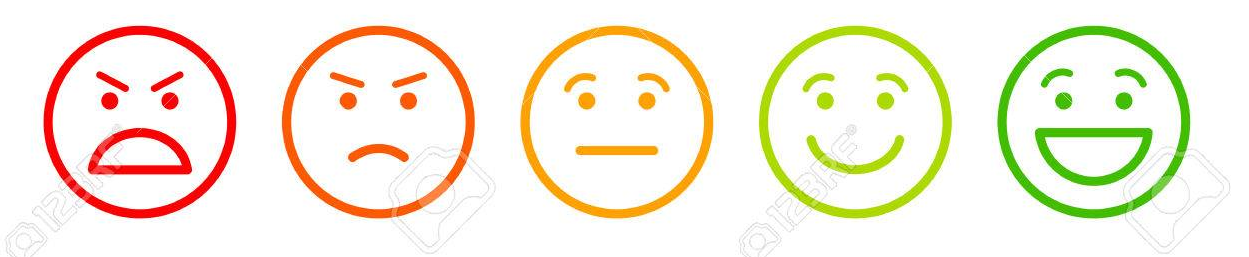** | | | | |
| Mis interacciones con el entorno virtual me parecieron naturales |  |  |  |  |  |
| He podido examinar objetos desde múltiples puntos de vista |  |  |  |  |  |
| Identifiqué correctamente los sonidos producidos por el entorno virtual |  |  |  |  |  |
| Me he sentido involucrado por los aspectos visuales del entorno virtual |  |  |  |  |  |
| La sensación de moverse dentro del entorno virtual era convincente |  |  |  |  |  |
| Me sentí estimulado por el entorno virtual |  |  |  |  |  |
| Me sentí físicamente en forma en el entorno virtual |  |  |  |  |  |
| A cada paso, sabía qué hacer |  |  |  |  |  |
| Sentí que controlaba la situación |  |  |  |  |  |
| Estaba perdiendo el sentido del tiempo |  |  |  |  |  |
| Esta experiencia me dio una gran sensación de bienestar |  |  |  |  |  |
| Cuando pienso en la experiencia en el entorno virtual, siento emociones que me gustaría compartir |  |  |  |  |  |
| Durante el uso, pensé que los dispositivos de interacción (auriculares, gamepad y / o teclado) eran fáciles de usar |  |  |  |  |  |
| La duración de la experiencia me parece buena |  |  |  |  |  |
| La frecuencia (número de días) de la experiencia es apropiada |  |  |  |  |  |
| Lo volvería a ver de nuevo mañana mismo |  |  |  |  |  |
| Me puse tenso en el entorno virtual |  |  |  |  |  |
| Disfruté tanto la experiencia que me siento con energía |  |  |  |  |  |
| Disfruté usando los dispositivos de interacción |  |  |  |  |  |
| Tuve mareos con los ojos abiertos durante mi interacción con el entorno virtual |  |  |  |  |  |
| Me sentí confiado seleccionando objetos en el entorno virtual |  |  |  |  |  |
| - En su opinión, ¿cuáles fueron los puntos positivos de su experiencia? |  |  |  |  |  |
|  |  |  |  |  |  |
| - En su opinión, ¿cuáles fueron los puntos negativos de su experiencia? |  |  |  |  |  |
|  |  |  |  |  |  |
| - ¿Tiene sugerencias para mejorar este entorno de realidad virtual? |  |  |  |  |  |
|  |  |  |  |  |  |
|  |  |  |  |  |  |
